# Neutrophil-to-Lymphocyte Ratio and Procalcitonin in Sepsis Patients: Do They Have Any Prognostic Significance?

**DOI:** 10.7759/cureus.62360

**Published:** 2024-06-14

**Authors:** Akash Singhal, Shruti Dubey, Shehtaj Khan, Reshma Tiwari, Saurabh Das, Reyaz Ahmad

**Affiliations:** 1 Critical Care Medicine, Max Super Speciality Hospital, Lucknow, Lucknow, IND; 2 Emergency Medicine, LN Medical College and Research Center, Bhopal, IND; 3 Critical Care Medicine, Artemis Hospital, Bhopal, IND; 4 Critical Care Medicine, Max Super Speciality Hospital, Shalimar Bagh, New Delhi, IND; 5 Pediatric Surgery, All India Institute of Medical Sciences, Bhopal, IND

**Keywords:** neutrophil-to-lymphocyte ratio, nlr, mortality, sepsis, procalcitonin

## Abstract

Introduction: Biomarkers like white blood cells, C-reactive protein, procalcitonin, and interleukin-1 are used in patients with sepsis for early diagnosis, differentiating various infections, making decisions to start antibiotics and evaluate their response, and to prognosticate morbidity and mortality. Despite the availability of these biomarkers, the prognosis of patients with sepsis in the ICU remains poor. Hence, this study was carried out to test the efficacy of procalcitonin and neutrophil-to-lymphocyte ratio (NLR) to prognosticate mortality and morbidity in terms of incidence of organ dysfunction and length of ICU stay in sepsis patients.

Methods: In this prospective observational study, we measured NLR and procalcitonin at days one, three, and seven of sepsis patients and divided them into four groups: low NLR and high procalcitonin (LNHP), high NLR and high procalcitonin (HNHP), high NLR and low procalcitonin (HNLP), and low NLR and low procalcitonin (LNLP). Mortality at 28 days was noted as the primary outcome.

Results: Out of 85 patients included in the study, five were lost to follow-up. Although no statistically significant difference was found in the primary outcome between all four groups, regression analysis showed that rising NLR and procalcitonin values were associated with a significant increase in mortality.

Conclusion: Serial values of NLR and procalcitonin are more important in predicting severity in comparison to a single value at presentation and can be used as a prognostic marker in sepsis patients.

## Introduction

Despite progress over the past half-century in treating patients with sepsis, the incidence of infection, sepsis, and septic shock is still increasing [1,2]. In-hospital mortality of sepsis ranges from 28.3% to 41.1% in North America and Europe [1,3]. In India, the intensive care case pattern study reported a frequent prevalence of sepsis, with an intensive care unit (ICU) mortality rate of 34% [4]. Various biomarkers like white blood cells (WBCs), C-reactive protein (CRP), procalcitonin, and interleukin-1 (IL-1) are used in patients with sepsis for early diagnosis, differentiating infection and inflammation, differentiating bacterial and non-bacterial infections, making decisions to start antibiotics and evaluate their response, and to prognosticate morbidity and mortality [1,5,6]. Several studies have reported procalcitonin having better diagnostic and prognostic value as compared with CRP. It clearly distinguishes viral and bacterial infections [4,7], rises more rapidly when compared to CRP levels, and peaks within a very short time. Also, if the patient responds appropriately to the treatment, the level of procalcitonin returns to normal range faster than CRP [4,8]. The neutrophil-to-lymphocyte ratio (NLR) is a readily accessible biomarker calculated from complete blood count [9]. Some studies have proposed NLR as an independent predictor of poor survival in various clinical circumstances, such as in oncological patients, critically ill patients, cardiology patients, and patients with sepsis.

Even with the availability of these biomarkers, the prognosis of patients with sepsis in the ICU remains poor. Hence, the aim of this study was to test the efficacy of procalcitonin and NLR to prognosticate mortality and morbidity in terms of incidence of organ dysfunction and length of ICU stay in sepsis patients.

The objectives of this study were to study 28-day mortality (primary objective), Sequential Organ Failure Assessment (SOFA) scores, length of ICU stay, and seven-day mortality in sepsis patients.

## Materials and methods

Study design and setting

This prospective single-center study was conducted at Artemis Hospital, Gurugram, Haryana in northern India after obtaining approval (No. 504256) from the Artemis Health Sciences Institutional Ethics Committee (AHS-IEC). All patients presenting with sepsis who met the following inclusion and exclusion criteria during a one-year period (April 2020 to April 2021) were enrolled in the study.

Inclusion criteria included all patients aged more than 18 years giving informed consent who presented with sepsis, as diagnosed by the Systemic Inflammatory Response Syndrome (SIRS) criteria.

Exclusion criteria included patients with immunosuppressive diseases (cancer, HIV infection) or on immunosuppressive therapy and patients who were already in ICU for >48 hours and became septic secondarily (viral and fungal sepsis), those who come from outside diagnosed as sepsis, and those who did not gave consent for enrollment in the study.

NLR was calculated as a ratio of circulating neutrophil and lymphocyte counts. The normal ranges for leukocytes in our laboratory are 2.0-6.5 x109/L for neutrophil count and 1.0-3.0 x109/L for lymphocyte count. Normal procalcitonin was taken as <0.05 ng/ml value. There was no potential source of bias in this study.

After collecting data, the study group was divided into four subgroups on the basis of NLR (high > 10 and low < 10) and procalcitonin (high > 9 ng/ml and low < 9 ng/ml) as follows: HNLP: patients with sepsis who had high NLR and low procalcitonin; LNHP: patients with sepsis who had low NLR and raised procalcitonin; HNHP: patients with sepsis who had both high NLR and raised procalcitonin; LNLP: patients with sepsis who had low NLR and low procalcitonin.

The following variables were observed from the time of administration until seven days (at time intervals of one, three, and seven days) in all groups: age, sex, presenting complaints, initial diagnosis at presentation, co-morbidities, diagnosis at discharge, Acute Physiology and Chronic Health Evaluation (APACHE) II score at 24 hours, investigations done in terms of total leukocyte count (TLC), differential leukocyte count (DLC), procalcitonin, SOFA score, days of mechanical ventilation, total days in ICU, and patient outcomes at seven days and 28 days (in ICU or on telephonic follow up). The primary outcome was defined as death within 28 days after admission to the ICU.

Statistical analysis

Statistical analysis was done using Microsoft Excel (Microsoft Corporation, Redmond, WA) and SPSS (IBM Corp., Armonk, NY) version 21.0.0 and data were expressed as median, absolute numbers, and percentages. Data were analyzed with Kruskal-Wallis and chi-square tests for statistical significance. A p-value less than 0.05 was considered significant. Patients lost to follow-up were excluded from the study. Regression analysis of NLR and procalcitonin values was also done.

## Results

**Figure 1:**
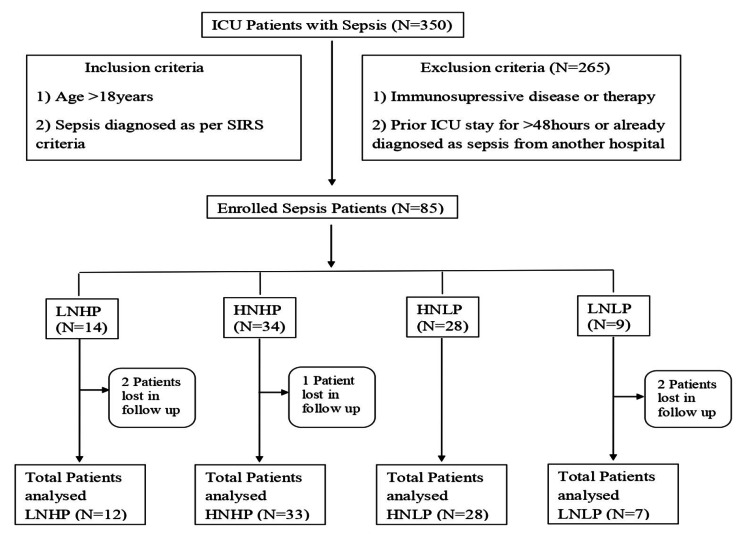
Distribution of patients The figure shows enrolled patients (N = number of patients) after applying inclusion and exclusion criteria and their distribution in four groups. HNLP: high neutrophil-to-lymphocyte ratio and low procalcitonin; LNHP: low neutrophil-to-lymphocyte ratio and high procalcitonin; HNHP: high neutrophil-to-lymphocyte ratio and high procalcitonin; LNLP: low neutrophil-to-lymphocyte ratio and low procalcitonin; SIRS: Systemic Inflammatory Response Syndrome.

A total of 350 patients came to the critical care department during the study period of one year with presenting complaints of fever and respiratory, urinary, and abdominal infections. SIRS criteria were applied to the patients coming with sepsis. Out of 350 patients, 265 were excluded from the study (Figure 1).

Of these 265 patients, 106 (40%) patients were those with a history of immunosuppressive disease or on immunosuppressive therapy, 90 (33%) patients were those coming from another hospital with diagnosed sepsis or who were already in ICU for >48 hours getting sepsis secondarily, 69 (26%) patients were excluded due to other reasons like consent not given by patient/unavailability of the biomarker in ED on the arrival of the patient.

In the study, 85 patients were enrolled after applying inclusion and exclusion criteria. Patients were divided into four groups, namely, low NLR with high procalcitonin (LNHP, N = 14), high NLR and high procalcitonin (HNHP, N = 34), high NLR with low procalcitonin (HNLP, N = 28), and low NLR and low procalcitonin (LNLP, N = 9). Five (4.25%) patients could not be analyzed in the study as they were lost in follow-up. Among these, two patients were from the LNHP group, one was from the HNHP group, and two were from the LNLP group. Finally, 80 patients were analyzed in the study. Out of 80 patients, 42 (52.5%) were females and 38 (47.5%) were males. The age of the patients ranged from 18 to 85 years and the median age was 38 years in the LNHP group, 68 years in the HNHP group, 69 years in the HNLP group, and 41 years in the LNLP group, as depicted in Table 1. The median age in the HNLP and HNHP groups is significantly higher than that of the LNHP and LNLP groups (p-value = 0.018). BMI values were not found to be statistically significant (Table 1).

**Table 1:** Demographic variables The table shows the median age in years and median BMI in kg/m^2^ with interquartile range (IQR) in all groups. P-value < 0.05 is considered significant (Kruskal-Wallis test). HNLP: high neutrophil-to-lymphocyte ratio and low procalcitonin; LNHP: low neutrophil-to-lymphocyte ratio and high procalcitonin; HNHP: high neutrophil-to-lymphocyte ratio and high procalcitonin; LNLP: low neutrophil-to-lymphocyte ratio and low procalcitonin.

Demographic variables	LNHP (N = 12)	HNHP (N = 33)	HNLP (N = 28)	LNLP (N = 7)	P-value
Age (years)	38 (34-69.25)	68 (51.5-71)	69 (51.5-77.5)	41 (27–69)	0.018
BMI (kg/m^2^)	24.78 (22.8-27.36)	26 (23-29)	26 (23-28)	25 (22-27)	0.488

Co-morbidities like diabetes mellitus, hypertension, chronic kidney disease, and coronary artery disease were seen across all groups and the difference was insignificant.

At the hospital, 46 (58%) patients presented with septic shock, 20 (25%) presented with severe sepsis, and the remaining 14 (17%) had sepsis. At discharge, 37 (47%) patients had pneumonia, 16 (21%) patients had UTI, five (6%) patients had meningitis, 10 (13%) patients had abdominal infections, and 10 (13%) patients presented with complaints of pyrexia of unknown origin.

The median APACHE II score at presentation was 16 in the LNHP group, 18 in the HNHP group, 12 in the HNLP group, and 10 in the LNLP group. The statistical analysis shows a significant difference (p-value = 0.016) between the groups with the highest value in HNHP, followed by LNHP > HNLP > LNLP.

Median SOFA scores at the presentation were 6 in the LNHP group, 6 in the HNHP group, 5 in the HNLP group, and 6 in the LNLP group. No statistically significant difference was found, as depicted in Table 2.

**Table 2:** Clinical disease severity score The table shows the median with interquartile range (IQR) and p-value of APACHE II and SOFA scores at presentation in all four groups. P- value < 0.05 is considered statistically significant (chi-square test). HNLP: high neutrophil-to-lymphocyte ratio and low procalcitonin; LNHP: low neutrophil-to-lymphocyte ratio and high procalcitonin; HNHP: high neutrophil-to-lymphocyte ratio and high procalcitonin; LNLP: low neutrophil-to-lymphocyte ratio and low procalcitonin; APACHE: Acute Physiology and Chronic Health Evaluation; SOFA: Sequential Organ Failure Assessment.

Scoring system at presentation	LNHP (N = 12)	HNHP (N = 33)	HNLP (N = 28)	LNLP (N = 7)	P-value
APACHE II score	16 (7.25-20.75)	18 (15-21)	12 (8.25-17.75)	10 (7-18)	0.016
SOFA score	6 (4-10.5)	6 (6-8)	5 (4-6)	6 (4-7)	0.086

Primary outcome

NLR and serum procalcitonin levels were measured on arrival, followed by repeat markers on day three and day seven. At 28 days in the LNHP group, eight (66.7%) were alive, but four (33.3%) patients died. In the HNHP group, we saw similar findings with a larger size, i.e., 22 (66.7%) patients were alive and 11 (33.3%) patients died, and in the HNLP group, 25 (89.3%) remained alive and three (10.7%) died. In the LNLP group, all patients survived. The statistical analysis showed no significant difference in all four groups, with a p-value of 0.063 (Table 3).

**Table 3:** Primary outcome in terms of mortality at 28 days The table shows the number (n (%)) of alive and dead patients at 28 days of presentation in all four groups. P-value < 0.05 is considered statistically significant (chi-square test). HNLP: high neutrophil-to-lymphocyte ratio and low procalcitonin; LNHP: low neutrophil-to-lymphocyte ratio and high procalcitonin; HNHP: high neutrophil-to-lymphocyte ratio and high procalcitonin; LNLP: low neutrophil-to-lymphocyte ratio and low procalcitonin.

Primary outcome		LNHP (N = 12)	HNHP (N = 33)	HNLP (N = 28)	LNLP (N = 7)	P-value
Mortality at 28 days	Survival	8 (66.7%)	22 (66.7%)	25 (89.3%)	7 (100%)	0.063
	Death	4 (33.3%)	11 (33.3%)	3 (10.7%)	0	

Secondary outcome

Serial SOFA scores were done on day three and day seven from admission for comparison of organ dysfunction in each group. The trend of SOFA scores on day three and day seven was significantly higher in the HNHP and LNHP groups.

Median ventilator days were five in the LNHP group, five in the HNHP group, one in the HNLP group, and two in the LNLP group, respectively. After applying the Kruskal-Wallis test, there was no statistically significant difference between the four groups.

The median days of ICU stay in the LNHP, HNHP, and HNLP groups were 10 while only seven days in the LNLP group. The statistical analysis showed no significant difference (p-value = 0.160) (Table 4).

**Table 4:** Secondary outcome The table shows the SOFA scores on days one, three, and seven, total days on a ventilator, and total days in the ICU with interquartile range (IQR) in all four groups. P-value <0.05 is considered statistically significant (Kruskal-Wallis test). HNLP: high neutrophil-to-lymphocyte ratio and low procalcitonin; LNHP: low neutrophil-to-lymphocyte ratio and high procalcitonin; HNHP: high neutrophil-to-lymphocyte ratio and high procalcitonin; LNLP: low neutrophil-to-lymphocyte ratio and low procalcitonin; SOFA: Sequential Organ Failure Assessment.

Secondary outcomes	LNHP (N = 12)	HNHP (N = 33)	HNLP (N = 28)	LNLP (N = 7)	P-value
SOFA score (day 1)	6 (4-10.5)	6 (6-8)	5 (4-6)	6 (4-7)	0.086
SOFA score (day 3)	6 (3.25-8.75)	4 (2-8.5)	3 (2-4)	2 (1-3)	0.004
SOFA score (day 7)	4 (1-7)	2 (1-7.5)	2 (0-3)	1 (0-2)	0.036
Total days of ventilator	5.5 (0.5-10)	5 (0-8.5)	1 (0-5.75)	2 (0-3)	0.160
Total days in ICU	10 (7.25-14)	10 (8-16.5)	10 (7-15.75)	7 (5-9)	0.065

We also saw mortality at seven days in all four groups. In the LNHP group, 12 (100%) patients survived. In the HNHP group, 33 (93.9%) patients survived and two (6.1%) were non-survivors. In the HNLP and LNLP groups, 100% of patients survived at seven days, i.e., 28 and seven, respectively. This was found to be statistically insignificant (p-value = 0.404).

We further observed NLR and procalcitonin on day three and day seven in each of the groups individually at 28 days in patients who survived and in patients who did not survive. In all four groups, values of procalcitonin and NLR decreased significantly in patients who survived. Since there was no death in the LNLP group and only three non-survivors in the HNLP group, they could not be included in our analysis to see the progression. Whereas in both LNLP and HNLP groups, when procalcitonin and NLR were seen on day three and day seven, they were found to be statistically significant in the case of both NLR and procalcitonin (Table 5).

**Table 5:** NLR and procalcitonin values on day one, day three, and day seven The table shows the NLR and procalcitonin (Procal) median values with interquartile range (IQR) on days one, three, and seven in patients who survived and who died in all four groups with p-values. P-value < 0.05 is considered statistically significant (chi-square test). NLR: neutrophil-to-lymphocyte ratio; HNLP: high neutrophil-to-lymphocyte ratio and low procalcitonin; LNHP: low neutrophil-to-lymphocyte ratio and high procalcitonin; HNHP: high neutrophil-to-lymphocyte ratio and high procalcitonin; LNLP: low neutrophil-to-lymphocyte ratio and low procalcitonin.

Parameter	LNHP (N = 12)			HNHP (N = 33)			HNLP (N = 28)		LNLP (N = 7)	
	Survival	Death	P-value	Survival	Death	P-value	Survival	Death	Survival	Death
NLR D1	7.5 (6.4-8.3)	7 (3.9-8.5)	0.933	27 (18.1-46.0)	16.0 (11.5-30.0)	0.036	20.0 (13.8-29.5)	11.5	6.6 (5.7-9.0)	-
NLR D3	6.2 (4.9-7.3)	10.0 (9.4-10.8)	0.004	7.45 (5.6-20.05)	18.8 (16.0-45.5)	0.002	8.1 (4.3-17.0)	8.2	4 (2.6-7.0)	-
NLR D7	4.15 (2.5-5.7)	10.5 (5.55-16.65)	0.048	3.7 (2.68-4.55)	15 (9.8-31.0)	<0.001	4.6 (4.15-7.35)	15.0	2 (0.9-3.5)	-
Procal D1	13 (11.3-21.5)	13 (6.75-18.5)	0.683	20.7 (13.9-40.5)	14 (11-32)	0.082	6 (4-6.35)	6.0	6 (3.57-7.0)	
Procal D3	5 (4-6.8)	20.5 (17.7-22.8)	0.048	7.8 (5.75-14.5)	12 (8.5-30)	0.048	1.98 (1-2.68)	5.0	1 (0.5-2.0)	
Procal D7	1 (0.6-1.75)	16 (9-20.75)	0.048	1.0 (0.15-4.0)	16.0 (8.26-28)	<0.001	0.150 (0.075-0.50)	4.0	0.15 (0.05-0.15)	

Regression analysis

On regression analysis of NLR values, the following observations were made, as shown in Table 6.

**Table 6:** Regression analysis of NLR values The table shows regression coefficient (B), standard error (SE), Wald statistic and its significance level (Sig.), degree of freedom (df), and exponentiated beta (Exp(B)) values. NLR: neutrophil-to-lymphocyte ratio.

Variables in the equation						
	B	SE	Wald	df	Sig.	Exp(B)
NLR	0.189	0.054	12.399	1	0.000	1.208

The positive value of the regression coefficient (B) suggests that the increased value of NLR is associated with increased mortality.

The Wald statistic and its significance level (Sig.) show that the relationship between high NLR values and mortality is statistically significant (p-value < 0.05). This means the observed effect is unlikely due to chance.

The exponentiated beta (Exp(B)) is 1.208. This signifies that a one-unit increase in the NLR values is associated with a 1.208-fold increase in mortality. In simpler terms, for every unit increase in the effect of the treatment, the risk of NLR values goes up by 20.8%.

On regression analysis of procalcitonin values, the following observations were made, as shown in Table 7.

**Table 7:** Regression analysis of procalcitonin values The table shows regression coefficient (B), standard error (SE), Wald statistic and its significance level (Sig.), degree of freedom (df), and exponentiated beta (Exp(B)) values.

Variables in the equation						
	B	SE	Wald	df	Sig.	Exp(B)
Procalcitonin	0.194	0.052	13.753	1	0.000	1.215

The positive value of the regression coefficient (B) suggests that a higher procalcitonin value is associated with increased mortality.

The Wald statistic and its significance level (Sig.) show that the relationship between procalcitonin value and mortality is statistically significant (p-value < 0.05). This means the observed effect is unlikely due to chance.

The exponentiated beta (Exp(B)) is 1.215. This signifies that a one-unit increase in procalcitonin value is associated with a 1.215-fold increase in the mortality of the event. In simpler terms, for every unit increase in procalcitonin value, the risk of mortality occurring goes up by 21.5%.

## Discussion

Severe sepsis and septic shock represent one of the oldest and most pressing problems in medicine. Sepsis when associated with organ dysfunction or shock is a major contributor to poor outcomes. With advances in intensive care, increased awareness, and dissemination of evidence-based guidelines, clinicians have taken large strides in reducing the risk of imminent death associated with sepsis [10].

The potential clinical usefulness of some innovative biomarkers has been discussed in the diagnosis, staging, and monitoring of sepsis, and these biomarker-guided strategies may allow more refined risk stratification and lead to improved patient care and outcomes [11,12].

The management of sepsis is quite challenging. The earlier and more rapid response toward sepsis results in a higher chance of survival. This current study presents our experience of procalcitonin and neutrophil and lymphocyte ratio as an earlier prognosticating marker of sepsis along with dynamical changes and their effects on mortality.

Demographic profile of the study population

In the present study, 80 patients were enrolled, of which 42 (52.5%) were females and 38 (47.5%) were males. A total of 51 (63.75%) patients were more than 60 years of age. This is in concordance with various studies [13-15]. The median age of patients in the HNHP and HNLP groups was 68 years and 69 years, respectively, and 38 years in the LNHP group and 41 years in the LNLP group. After applying the Kruskal-Wallis test, statistical significance was found among all four groups. Also, the median for age was higher in groups with higher NLR levels irrespective of procalcitonin levels. This is in concordance with a study done by Fest et al., which states that higher age is associated with higher NLR (the Rotterdam study) [16].

Clinical profile of the study population

On arrival, 46 (58%) patients were in septic shock, 20 (25%) patients were in severe sepsis, and only 14 (17%) patients were in sepsis. This is in agreement with the findings of a study done by Ginde et al. where 325 (38%) patients had sepsis, 137 (16%) had severe sepsis without shock, and 393 (46%) had septic shock [17]. Sudhir et al. in their study state otherwise, in which, out of 100 patients, 52 (52%) presented with sepsis, 25 (25%) with severe sepsis, and 23 (23%) presented with septic shock [18]. A total of 41 (35.3%) patients had septic shock in a study done by Vucelic et al. in Croatia [19]. We had a higher number of patients with septic shock than any other studies. This came most likely due to the fact that other studies were done before the COVID-19 pandemic arrived. Our study period was from April 2020 to April 2021, when the COVID-19 pandemic was at its peak, and hence, more patients were sick at the time of presentation. The most common diagnosis in 37 (47%) patients was pneumonia, and UTI came second with 16 (21%) patients, followed by abdominal sepsis in 10 (13%), pyrexia of unknown origin in 10 (13%), and meningitis only in five (6%) patients.

In our study, mortality due to sepsis was seen in 18 (22.5%) patients, which was consistent with the reported wide range (18% to 56%) of overall mortality due to sepsis [20,21]. Also in our study, mortality increased with the severity of sepsis, which is in concordance with that reported in previous studies [21,22].

Scores

In our study, the APACHE II score was tested at presentation and was higher in the HNHP and LNHP groups when compared to the LNLP and HNLP groups. This was analyzed using the Kruskal-Wallis test and was found statistically significant (p-value = 0.016). This is in concordance with a study recently done by Songlin Su [23]. They showed that procalcitonin and CRP levels were the highest in patients with APACHE II scores > 20, followed by those with scores of 11-20 points and those with scores of 0-10 points, respectively (P < 0.05) [23]. In a study done by Jain et al., the baseline severity of illness scores, including APACHE II, SOFA, and the Simplified Acute Physiology Score II, was significantly higher among non-survivors than survivors. They further did a multivariate analysis in which only the APACHE II score at the time of admission was found to be an independent predictor of mortality (p-value = 0.005) [24].

In our study, the SOFA scoring was done at the presentation. No significant difference between SOFA scores was found across all four groups.

Primary outcome

We followed patients in-hospital or telephonically on the 28th day. There was no significant correlation between mortality, NLR, and serum procalcitonin levels at the time of admission (p-value = 0.063). There have been various studies that consider high procalcitonin and NLR at admission as predictors of mortality [9,24-26]. However, contrasting results have been reported in other studies that found no significant correlation, just like us [13,27].

Ni et al. could not find any positive correlation between NLR done at admission and in-hospital survival/mortality, which might be due to the fact that they only recorded the NLR at admission while it would have been more rational to monitor dynamic NLR changes [13].

Secondary outcome

SOFA score was tested at the presentation and then on days three and seven. Both the LNHP and HNHP groups had a higher median value when compared to the other two groups, i.e., HNLP and LNLP groups. This was found to be statistically significant. Jain et al. found similar results and concluded that SOFA score at 48 hours of admission correlated with high serum procalcitonin levels [24].

No significant difference was noted across the groups in the length of ICU stay and total days on the ventilator.

In all four groups, after applying the chi-square test in our study, when we looked at survivors vs. non-survivors at seven days, there was no statistical difference, i.e., a p-value of 0.404 among all four groups. There were two deaths noted in the HNHP group. One was due to urosepsis and another was due to lower respiratory tract infection. Interestingly both patients had age > 60 years, diabetes mellitus, hypertension, APACHE score > 18, SOSA score > 6, and high NLR (>10) and high procalcitonin (>9). The reason for us getting insignificant results might be due to the fact we have excluded patients with an immunosuppressive disorder/patients on immunosuppressive drugs, patients coming with fungal etiology, or those with secondary sepsis who are at greater risk of early death or decompensation. Daviaud et al. studied the timing and cause of death in septic shock and found independent determinants of early death were age, malignancy, diabetes mellitus, no pathogen identification, and initial severity [15].

When we analyzed NLR serially on day one, followed by day three and day seven, we found that NLR decreased significantly in survivors in all groups. In non-survivors, the number of deaths in the HNLP group was insignificant and in LNLP there were no deaths. This decrease in serial NLR done on day three and day seven in survivors was found to be statistically significant (p-value of 0.004 on day three and p-value of 0.048 on day seven in the LNHP group and p-value of 0.002 and <0.001 on day three and day seven, respectively, in the HNHP group). Similar results were reported by Lee et al., who concluded that the progression of NLR is a better predictor of outcomes in sick patients [28]. The reason was also explained nicely by Lee et al. in their study [28].

In our study, when we assessed procalcitonin on days one, three, and seven, we found that in all groups, the procalcitonin in series decreased significantly in patients who survived. In the HNHP and LNHP groups, procalcitonin did not decrease significantly or increase in patients who did not survive. This corroborates with the MOSES study done by Schuetz et al., in which they state that 28-day all-cause mortality was two-fold higher when procalcitonin did not show a decrease of more than 80% from baseline to day four (20% vs. 10%, p = 0.001). Also, they confirmed procalcitonin as an independent predictor in Cox regression analysis (p < 0.009) after adjusting for demographics, APACHE II, ICU residence on day four, sepsis syndrome severity, antibiotic administration time, and other relevant confounders [29]. This is also in concordance with a study done by Jain et al., in which the level of procalcitonin decreased significantly in survivors over 28 days. The median of procalcitonin fell from 5.4 ng/mL on day one to 3.1 ng/mL on day seven (p = 0.002) to 0.1 ng/mL on day 28 (p = 0.01) [24]. Also, in a study done by Giamarellos-Bourboulis et al., procalcitonin was gradually diminished over time with the resolution of the syndrome, while it was sustained in the same or more augmented levels upon worsening [30].

In our research, the study population was a large, diverse group of critically ill adult patients with sepsis, severe sepsis, and septic shock admitted to ICU. We evaluated the role of serum procalcitonin and NLR as predictors of sepsis and explored the prognostic accuracy of these two parameters in our clinical practice.

Limitations of the study

It was a single-center study with a relatively small sample size, which may limit the generalizability of the findings. In our study, no blinding was done and we did not review cultures and antibiotic response in the case of survivors and non-survivors in all groups. Excluding patients with immunosuppressive diseases or immunosuppressive therapy may overlook the prognostic significance of NLR and procalcitonin in a broader spectrum of sepsis patients. Also, we did not take into account any medical or surgical intervention done during the hospital stay, such as renal replacement therapy, intercostal drain placement, or non-invasive ventilator support. Therefore, the decrease in NLR and procalcitonin cannot be attributed only to disease progression alone but also to treatment response, which in our study was not taken into account.

## Conclusions

The present study showed that initial NLR and procalcitonin values do not predict all-cause 28-day mortality. Serial values of both biomarkers may be more important in predicting severity in comparison to a single value at presentation. Further studies are required to clarify these results and to assess the utility of procalcitonin and NLR as predictors of mortality and treatment response in sepsis, severe sepsis, and septic shock patient populations.
